# M. Louis on Typhoid Fever

**Published:** 1842-11

**Authors:** 


*M. Louis on Typhoid Fever.*—We observe by the French journals that a new edition of M. Louis' well known work on this subject has recently been published in Paris. Its title deserves notice: 'Recherches Anatomiques, Pathologiques et Therapeutiques sur la Maladie connue sous les Noms de Fievre Tyhoide, Putride, Adynamique, Ataxique, Bilieuse, Muqueuse, Gastro-enterite, Enterite Folliculeuse, Dothinenterite, &c., comparée avec les Maladies Aigues les plus ordinaries!' Does not this alone indicate how bewildered the ideas of medical men must still be on the all important subject of fever? We had almost hoped that M. Louis would by this time have found reason to modify his opinion as to the seat or proximate cause of typhoid fever being in the intestinal canal; but we find that we are mistaken. He still maintains, and even in more decided terms than ever, that 'the anatomical character of the typhoid affection (a very stupid phrase) consists in a morbid alteration of the intestinal, chiefly the Peyerian glands; and, also, that any secondary lesions are very usually of an inflammatory nature.'

This doctrine is a most fallacious, and unfortunately at the same time a most hurtful, one. While we admit that the intestinal lesions seem, from all accounts, to be of much more frequent occurrence in fever as it occurs in France, than as it is seen in this country, we most confidently deny that they are uniform or constant even in the former case—at least if we can trust the testimony of such men as Chomel and Andral. But even admitting the frequency of intestinal lesions in fever, does it necessarily follow that they are the consequences of simple inflammatory action, as asserted by Broussais, Louis, Bouillaud, and others of this school? Do we observe the same phenomena on dissection in typhus as are found in fatal cases of genuine enteritis? Assuredly not. The patchy redness in different parts of the canal, the tumefied and softened condition of the mucous and submucous tissues, the enlargement, ulceration, and even sphacelation of the follicular glands, &c., cannot surely be admitted as the legitimate evidences of a pure phlegmasia. They might rather be regarded as the results of the irritation of unhealthy acrid secretion on the mucous surface than as the primary and essential phenomena of the disease. But without going so far as this, we have no hesitation in asserting that the intestinal lesions, however frequent they may be in the fevers of Paris, can only be viewed as one of the changes induced by the general disease of the system. To apply the term of *enteritis* to such lesions is not only incorrect in theory, but most pernicious in practice. It has been from the too general adoption of this dogma by the present generation of medical men in France, that so many and so serious errors have been committed by them in the treatment of their patients. When we read the grave discussions that are frequently agitated in the Academy of Medicine of Paris, on the results of some novel mode of treating typhoid fever, what are we to expect from the young surgeons and physicians who are annually drafted away either to the country, or perhaps into the army and navy, immediately after having had their heads crammed with the 'nouvelle doctrine' as taught by the professors of the physiological school!

Part of M. Louis' work is taken up with showing that 'jail-typhus and typhoid fever are identically the same disease.' What rational man ever doubted it? But such are the vague opinions of French medical men on the subject of fever, that M. Louis deems it necessary to devote an entire chapter to prove a position, which has been recognized as an acknowledged truth by British practitioners ever since, and indeed long before the days of Huxham. But then it is at the same time well known that almost every epidemic of fever, whether it appears in jails or other crowded buildings, or in the lanes and dirty alleys of a city, exhibits some peculiarity or other by which it may be characterised; and yet no sensible physician would dream of designating one epidemic as cephalitis, another as bronchitis, a third as gastro-enteritis, merely because the predominant symptoms may be at different times seated in the head, chest, or abdomen. Until medical men can be induced to carry their thoughts beyond the more obvious lesions discoverable on the dissection of patients who fall a victim to typhus fever, they will ever remain involved in a labyrinth of perplexities, which will only be increased by a more extensive acquaintance with the disease. A calm unprejudiced examination of the history of typhus must lead every one, we are inclined to believe, to the conviction that it is the result of an invisible atmospheric agent—call it miasm, malaria, matter of contagion, or what you will—on the human body, acting primarily on the nervous system, and often rapidly absorbed into the system, thereby inducing a more or less decidedly vitiated state of the circulating fluids.

The lesions of the viscera, whether of the cranial, or of the thoracic or abdominal cavities, are all secondary or consecutive phenomena of the disease; their occurrence being attributable either to the contemporaneous influence of other atmospheric agencies, to the idiosyncrasy, habits, and previous state of health of the patients affected, or perhaps to the differences in the nature and degree of the aerial poison that has been introduced into the system. We must admit that, in taking this view of the question there are many points that cannot be demonstrated by actual proofs, and that the theory is essentially based upon a mere probability; but let us remember that there is, and ever will be, much in the history of these diseases, especially those of an epidemic and diffusive character, that must defy the scrutiny of human observation: we cannot *exhibit* the infectious poison of scarlet fever, or of measles, or the miasma which gives rise to intermittent or remittent fevers; and yet no one disputes their existence. It is therefore a very insufficient reply to be told that we cannot demonstrate our theory of the disease. Some modern French writers are constantly trying to make us believe that medicine is capable of being rendered an *exact* science, and that we must not admit any positions in our reasonings on disease but what can be proved by actual observation. This is a most foolish and dangerous doctrine. As in moral and political philosophy, so in medicine, there are no permanently fixed and unalterable rules which are inevitably true at all times and under all circumstances. The beauty of a moral precept or the expediency of a political maxim may charm the mind in theoretical speculation; but in our practical dealings with the world, we find, alas! that the line of conduct to be adopted must greatly depend upon the circumstances of each case —ever striving however to act on the principle of doing unto others as we wish them to do unto ourselves.

Dr. Landouzy, of Rheims, the reviewer of M. Louis' work in the French Medical Gazette, states that his own experience in the late epidemic of jail-typhus in that town confirms the opinion that it is analogous, if not identical, in nature with the typhoid fever of the metropolis, and he bases his opinion on the circumstance of the *constant* existence, in all cases and at all periods of the disease, of the intestinal lesions in both forms of the fever. Yet, strange to say, he informs us that in no case were there any symptoms of such lesions during the life: 'la diarrhée, la douleur abdominale, le meteorisme et le gargouillement ne se sont jamais rencontrés.' How can Dr. Landouzy, or any other sensible man, in the face of such a statement as this, continue in the belief that the disease, in which these symptoms were absent, was an inflammatory affection of the mucous coat of the bowels; in other words, that it was a 'gastro-enterite,' or 'dothinenterite.' He suggests, as a novel idea, that the difference in the symptoms of typhus fever in different times and seasons may be connected with some difference in the type or genus, as he calls it, of the disease, and very coolly remarks that 'the observations of authors on this point of pathology having in truth but little or no value until the 'beaux travaux' of M. Louis have become generally known, we must wait for new epidemics of typhus before we can offer a positive opinion upon the subject.'

Has Dr. Landouzy never heard of Sydenham or Huxham, or of Cullen, or of his own countryman Pinel, not to mention a century of other authors, who have all insisted upon this essential fact connected with the history of fevers—the differences in the character of the disease according to what has been demonstrated the 'medical constitution' of the season? Little is the wonder that the French are making discoveries in medicine almost every day: they seem to be utterly unaware of their own well expressed motto, 'nothing is so new as that which is forgot!'

The therapeutic portion of this edition of M. Louis' work has received, we are informed, 'd'immenses developpemens;' but the reviewer does not tell us particulars; all that he says is, that 'the author *seems* to prefer in the greater number of cases the use of evacuants to any other remedies.' The little word *seems* (semble) implies, if we are not much mistaken, a mighty deal in reference to M. Louis' mode of treatment. It is well known that this gentleman, however high he may be regarded as a morbid anatomist, cannot be appealed to as an authority on practical subjects. Some years ago, as we have noticed in a former article, he made the important discovery that bloodletting and blistering had little or no efficacy in subduing pneumonia! He has probably extended the application of this discovery to the treatment of fevers, and may have found out that by far the best mode of managing them is to do nothing.

In this respect his example offers a marked contrast with that of M. Bouillaud, a disciple of the same pathological school, whose 'grande decouverte' is the 'nouvelle formule' of bleeding 'coup sur coup,' so as to 'juguler' the disease at once!! But we trust that it is quite unnecessary to expose the fallacy of this gentleman's views at present, as we have so repeatedly of late years cautioned the English reader against the errors into which he is apt to lead the inexperienced by the bold and confident manner with which he proclaims his marvellous success.—*Med. Chir. Review.*

